# Selection of Highly Proliferative and Multipotent Meniscus Progenitors through Differential Adhesion to Fibronectin: A Novel Approach in Meniscus Tissue Engineering

**DOI:** 10.3390/ijms22168614

**Published:** 2021-08-10

**Authors:** Jasmijn V. Korpershoek, Margot Rikkers, Tommy S. de Windt, Marianna A. Tryfonidou, Daniel B. F. Saris, Lucienne A. Vonk

**Affiliations:** 1 Department of Orthopedics, University Medical Center Utrecht, 3584 CX Utrecht, The Netherlands; j.v.korpershoek-3@umcutrecht.nl (J.V.K.); M.Rikkers-2@umcutrecht.nl (M.R.); T.S.deWindt-2@umcutrecht.nl (T.S.d.W.); Saris.Daniel@mayo.edu (D.B.F.S.); 2 Department of Clinical Sciences, Faculty of Veterinary Medicine, Utrecht University, 3584 CL Utrecht, The Netherlands; M.A.Tryfonidou@uu.nl; 3 Department of Orthopedic Surgery and Sports Medicine, Mayo Clinic, Rochester, MN 55905, USA; 4 Department of Reconstructive medicine, University of Twente, 7522 NB Enschede, The Netherlands

**Keywords:** meniscus progenitors, MSCs, osteoarthritis, tissue-engineering regeneration

## Abstract

Meniscus injuries can be highly debilitating and lead to knee osteoarthritis. Progenitor cells from the meniscus could be a superior cell type for meniscus repair and tissue-engineering. The purpose of this study is to characterize meniscus progenitor cells isolated by differential adhesion to fibronectin (FN-prog). Human osteoarthritic menisci were digested, and FN-prog were selected by differential adhesion to fibronectin. Multilineage differentiation, population doubling time, colony formation, and MSC surface markers were assessed in the FN-prog and the total meniscus population (Men). Colony formation was compared between outer and inner zone meniscus digest. Chondrogenic pellet cultures were performed for redifferentiation. FN-prog demonstrated multipotency. The outer zone FN-prog formed more colonies than the inner zone FN-prog. FN-prog displayed more colony formation and a higher proliferation rate than Men. FN-prog redifferentiated in pellet culture and mostly adhered to the MSC surface marker profile, except for HLA-DR receptor expression. This is the first study that demonstrates differential adhesion to fibronectin for the isolation of a progenitor-like population from the meniscus. The high proliferation rates and ability to form meniscus extracellular matrix upon redifferentiation, together with the broad availability of osteoarthritis meniscus tissue, make FN-prog a promising cell type for clinical translation in meniscus tissue-engineering.

## 1. Introduction

The meniscus is a fibrocartilage structure in the knee that is predominantly composed of circumferentially orientated type I collagen fibres and low amounts of glycosaminoglycans (GAGs) surrounded by water. It plays an important role in the shock absorption, load transmission, and stability of the knee. Meniscus injuries can lead to knee pain, locking, and swelling and are highly disabling. The treatment of a meniscus injury is dependent on the location of the tissue damage, as the ability to heal differs between the inner and outer zones. The avascular inner zone is composed of chondrocyte-like cells and does not heal, while the vascularized outer zone has a fibrocartilage phenotype and some healing potential [1,2,3]. Therefore, meniscus tears in the outer zone of young patients can be successfully repaired, but overall, 66% of meniscus tears remain irreparable [4,5,6]. Meniscus tears unsuitable for repair are treated using (partial) meniscectomy with a seven-fold increase in the odds of developing osteoarthritis [7,8]. Currently, approaches for (stem) cell based therapies for meniscus repair and regeneration are emerging [9,10]. These therapies often employ multipotent mesenchymal stromal cells (MSC), but hypertrophy and osteogenesis are common drawbacks of these stem-cell like or signaling cells [11,12]. Results of the first clinical trial employing MSCs after meniscectomy are suboptimal [13] and there seems to be a paradigm shift towards the use of specific progenitor cells [14]. In the last decades, the existence of a progenitor cell population in healthy as well as osteoarthritic cartilage has been suggested [15,16,17]. Cartilage progenitor cells can be isolated by employing their differential adhesion to fibronectin (DAF) based on the high affinity for the fibronectin receptor [17]. Cartilage progenitor cells have high proliferative and multipotent capacity and increased chondrogenic differentiation potential compared to bone marrow MSCs [18,19], with a lower tendency for terminal hypertrophic differentiation [20]. Recently, the presence of meniscus progenitor cells has been suggested in rabbits [12,21] and humans [22,23,24]. Meniscus progenitor cells might be a therapeutic target for meniscus preservation and a promising cell type for meniscus tissue-engineering, especially due to the high availability of osteoarthritic tissue. However, meniscus progenitor cells are not thoroughly characterized, and unlike for articular cartilage, the DAF protocol has not been explored for the isolation of meniscus progenitors.

Therefore, the purpose of this study is to isolate progenitor cells from osteoarthritic menisci using DAF. To test the ability of DAF to select a progenitor population, the acquired cells (FN-prog) were compared to the total meniscus population (Men). Men and FN-prog were characterized according to the MSC guidelines by the International Society for Cellular Therapy (ISCT) [25]. Moreover, other progenitor characteristics, e.g., clonogenicity and proliferation, were assessed. Lastly, the potential of FN-prog for meniscus tissue engineering was compared to Men by assessing redifferentiation in pellet culture using gene expression, release, and deposition of GAGs and deposition of collagen.

## 2. Results

### 2.1. Selection of a Clonogenic Population from the Meniscus Inner and Outer Zone

**Figure 1:**
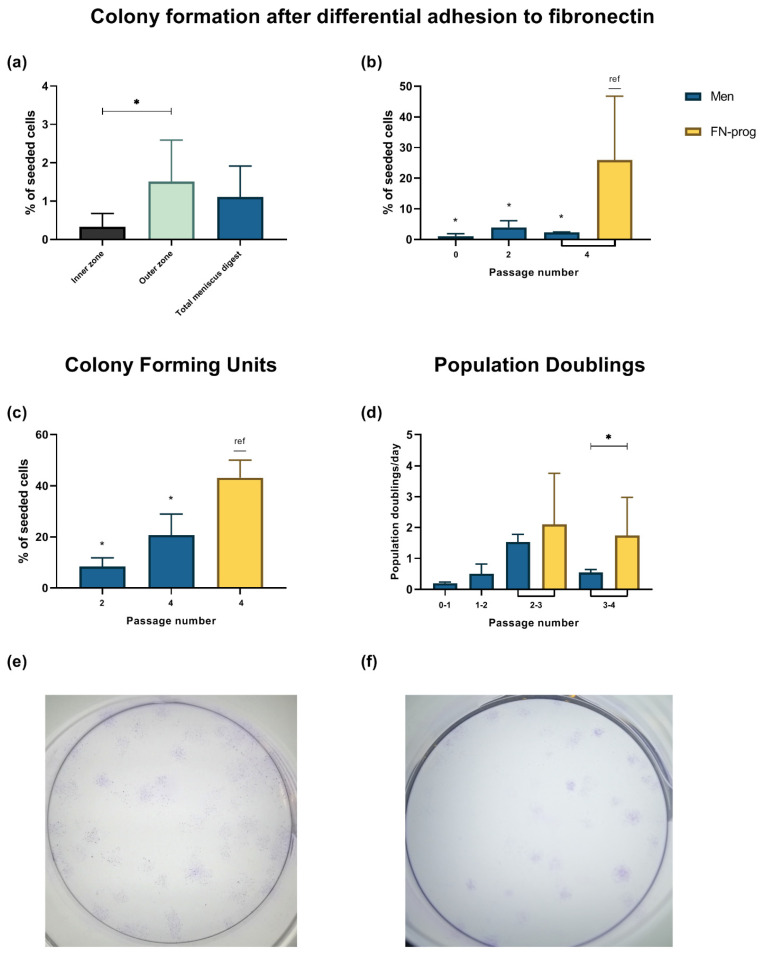
Colony formation after fibronectin selection of (**a**) inner zone, outer zone and total meniscus digest at passage 0 and (**b**) different passages of the total meniscus population (Men) and fibronectin selected cells (FN-prog), comparison between different passage numbers of the total meniscus population (Men) and fibronectin selected cells (FN-prog) in (**c**) colony forming units on culture dishes without fibronectin coating and (**d**) population doublings per day. Representative pictures of colonies formed after seeding (**e**) Men at 11 cells/cm^2^ or (**f**) FN-prog at 6 cells/cm^2^ both at passage 4, stained with crystal violet blue. * *p* < 0.05; ref, reference category.

Colony formation of the total meniscus as well as outer and inner zone digest was assessed after DAF. Of the total meniscus digest, 1.1 ± 0.8% of cells were clonogenic with affinity for fibronectin. Moreover, 0.3 ± 0.4% of the inner zone cells formed colonies, whereas 1.5 ± 1.1% of the outer zone cells formed colonies (Figure 1a). Fibronectin affinity of different passages of FN-prog and Men was assessed after 20 min adhesion to fibronectin (Figure 1b). Colony formation of FN-prog at passage 4 was higher than the formation of Men. To assess colony formation, regardless of fibronectin affinity, colony formation on culture dishes was also assessed. Again, colony formation of FN-prog at passage 4 was higher than that of Men (Figure 1c). Moreover, FN-prog retained their proliferative capacities, while the proliferation rate of Men diminished after the third passage (Figure 1d). Representative pictures of the colonies are shown in Figure 1e,f.

### 2.2. Expression of MSC Markers

**Figure 2:**
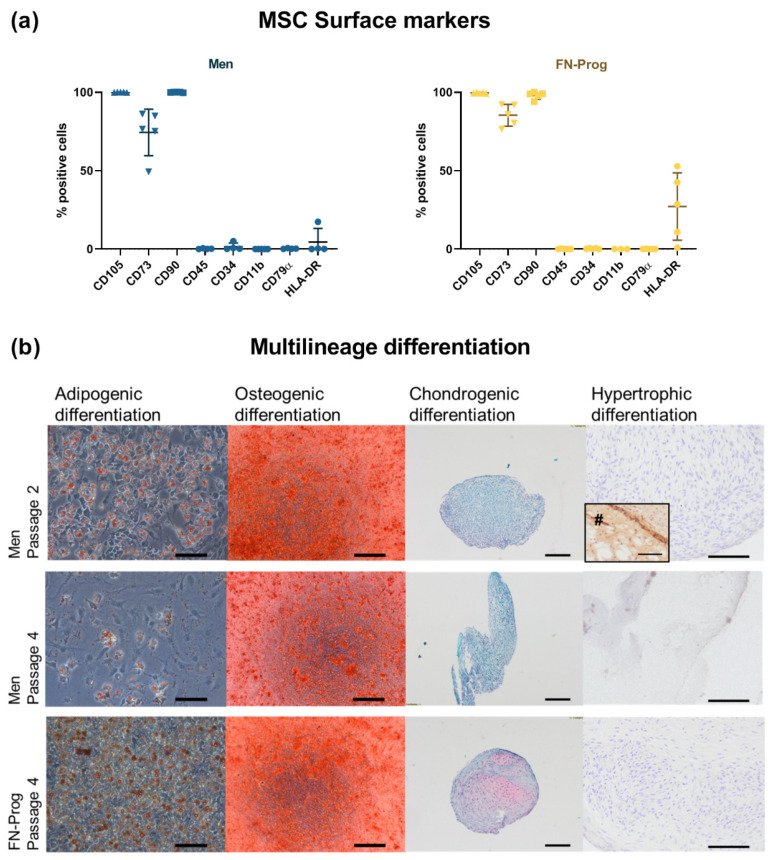
Characterization according to the MSC guidelines of the International Society for Cell & Gene Therapy [25] for (**a**) surface marker expression and (**b**) adipogenic (oil Red O staining), osteogenic (Alizarin Red staining), chondrogenic (Safranin-O/ Fast Green staining), hypertrophic (type X collagen immunohistochemistry) differentiation. *n* = 5 donors per group per condition. Donor-matched samples were used to culture the total meniscus population (Men) and fibronectin selected cells (FN-prog). #, bone marrow mesenchymal stromal cells passage 4 that were hypertrophically differentiated were used as positive control for type X collagen immunohistochemistry. Scale bars represent 100 µm.

Over 98% of FN-prog and Men expressed the surface markers CD90 and CD105. CD73 was expressed in 74 ± 15% of Men and 85 ± 7.0% of FN-prog. Negative markers CD45, CD34, CD79α, and CD11b were negative (<2% positive) in both FN-prog and Men. In four out of five donors, 11–53% of FN-prog expressed the HLA-DR receptor and therefore did not meet the ISCT criteria [25]. In one out of five Men donors, the HLA-DR receptor was expressed in 17% of the cells (Figure 2a).

### 2.3. Multilineage Potential

All cell populations showed oil Red O staining after three weeks of culture indicative of adipogenic differentiation. Similarly, all populations produced mineralized matrix upon osteogenic induction confirmed by Alizarin Red staining. After three weeks of pellet culture in chondrogenic medium, all 5 FN-prog donors showed GAG deposition as indicated by Safranin-O staining, whereas none of the donors showed GAG deposition in the total meniscus population at passage 2. Cells of the total meniscus population at passage 4 were not able to form or maintain a firm pellet up to four weeks and did not show GAG deposition. None of the populations showed hypertrophic differentiation when subjected to hypertrophic media [11] as assessed by type X collagen deposition, while the positive control of bone marrow derived MSCs was positive for type X collagen staining (Figure 2b).

### 2.4. Expression Profile after Monolayer Expansion

**Figure 3:**
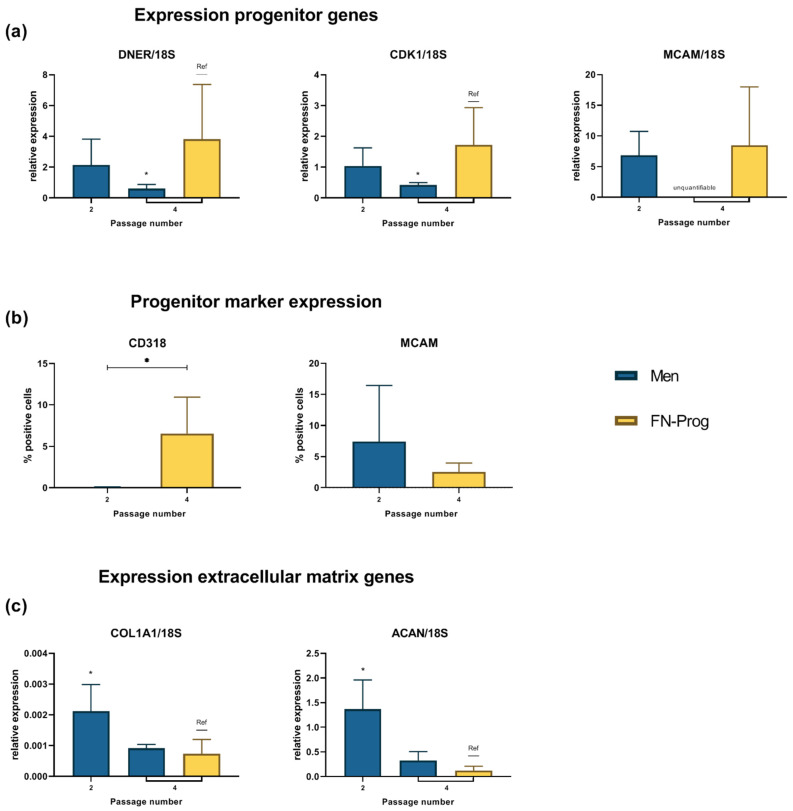
Expression of (**a**) genes associated with meniscus extracellular matrix production and (**b**) meniscus progenitor phenotype as measured by quantitative real-time PCR and (**c**) progenitor marker expression measured using flow-cytometry after monolayer expansion. *n* = 5 donors per group per condition. *ACAN*, aggrecan; *COL1A1*, collagen type I alpha 1 chain; *CDK1*, Cyclin-dependent kinase 1; *DNER*, Delta and Notch-like epidermal growth factor-related receptor; FN-prog, fibronectin selected cells; *MCAM*, Melanoma cell adhesion molecule; Men, total meniscus population * *p* < 0.05; ref, reference category.

Genes associated with a degenerative (Delta and Notch-like epidermal growth factor-related receptor (*DNER*)) or cartilage (Cyclin-dependent kinase 1(*CDK1*)) progenitor fate [22] were expressed relatively higher in FN-prog than Men at passage 4 (Figure 3a). CD318, a marker associated with degenerative meniscus progenitor cells, was expressed higher in FN-prog than Men (0.05 ± 0.06 vs. 6.5 ± 4.4) (Figure 3b). A meniscus progenitor fate [22] can be assessed using the pericyte marker Melanoma Cell Adhesion Molecule (*MCAM*) [26] (also known as CD146). Gene-expression of *MCAM* did not differ significantly between FN-prog at passage 4 and Men at passage 2 (Figure 3a). The expression of *MCAM* in Men passage 4 was below the limit for quantification using qPCR. Surface marker expression of *MCAM* as assessed by flow cytometry did not differ significantly between Men and FN-prog (7.5 ± 9.0 vs. 2.5 ± 1.5% positive cells) (Figure 3b). The expression of extracellular matrix genes collagen type I α1 chain (*COL1A1*) and aggrecan (*ACAN*) were lower in FN-prog than in Men (Figure 3c). The expression of collagen type II α1 chain (*COL2A1*) was detectable but not quantifiable.

### 2.5. Chondropermissive/Redifferentiation Culture

**Figure 4:**
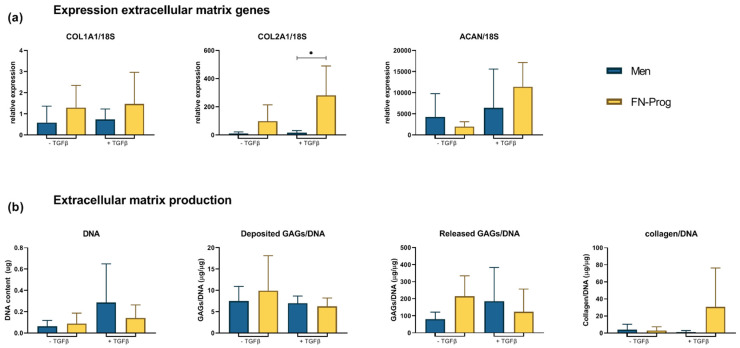
(**a**) Expression of genes associated with meniscus extracellular matrix production, and (**b**) DNA content, glycosaminoglycan deposition and release normalized for DNA content and collagen deposition measured by hydroxyproline assay, normalized for DNA content after 28 days of pellet culture in chondropermissive medium in absence or presence of TGF-β1. *n* = 5 donors per group per condition. *ACAN*, aggrecan; *COL1A1*, collagen type I α1 chain; *COL2A1*, collagen type II α1 chain; GAG, glycosaminoglycan; TGFβ, transforming growth factor beta 1; * *p* < 0.05.

**Figure 5:**
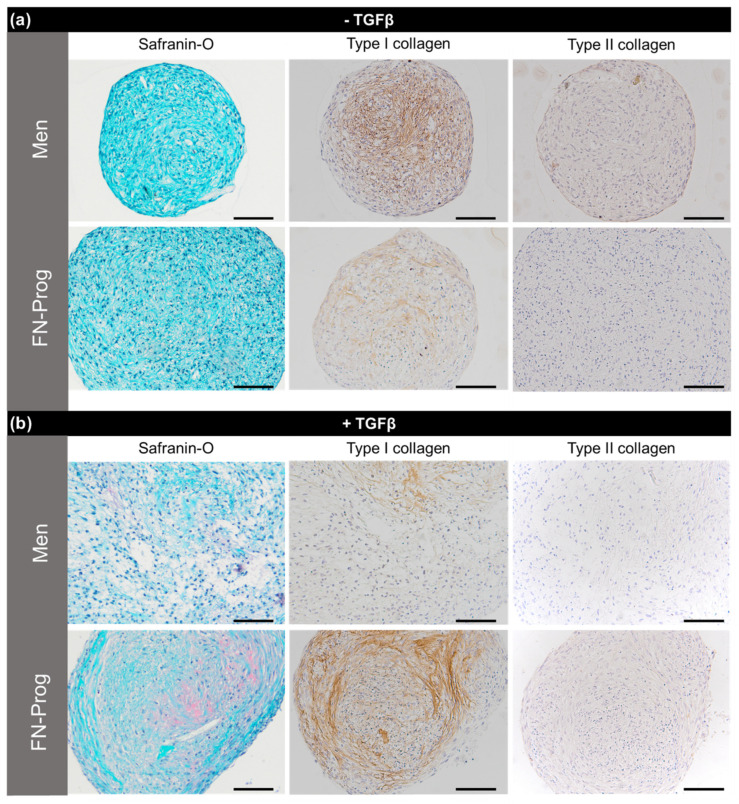
Representative sections of pellets cultured for 28 days in differentiation medium in (**a**) absence of TGF-β1 or (**b**) presence of TGF-β1. *n* = 5 donors per group per condition. Scale bars represent 100 µm.

After culturing both cell populations in pellets in chondropermissive medium for 28 days, gene expression of meniscus matrix genes, GAG production, and collagen and GAG stainings were analysed. *COL1A1*, *COL2A1*, and *ACAN* expression of FN-prog did not differ from Men. Upon addition of TGF-β1 to the chondropermissive medium, only *COL2A1* expression was higher in FN-prog compared to Men (Figure 4a). DNA content did not differ between groups. Similarly, total production of GAGs (deposition and release) was comparable. Collagen deposition in FN-prog seemed higher than Men but this did not reach statistical significance (*p* = 0.16) (Figure 4b). In the absence of TGF-β1, safranin-O staining indicative of glycosaminoglycan deposition was absent in all pellets. The deposition of type I collagen was more pronounced by pellets of Men cells compared to FN-prog. In the Men pellets, type I collagen was mainly found in the inner regions of the pellets, while for FN- prog it was distributed throughout the pellets, but in lower amounts. In the pellets that were cultured in presence of TGF-β1, safranin-O staining was positive in one donor of Men and all donors of the FN-prog. There was a low deposition of type I collagen by Men in some areas of the pellet, while FN-prog had deposited more type I collagen, that was located mostly towards the outer regions of the pellet. Type II collagen staining was absent or low in all groups (Figure 5).

## 3. Discussion

This is the first study to isolate meniscus progenitor cells through DAF and to characterize the obtained cell population according to the MSC criteria of the ISCT. We confirmed selection of a distinctive cell population which differs from the total meniscus population in terms of colony formation, proliferation, chondrogenic differentiation, and gene-expression. The advantages of this population in terms of expansion and redifferentiation potential make this a promising cell type for meniscus tissue engineering.

DAF has been used for isolation of progenitor cells from cartilage for almost two decades [17], but was never used for isolation of meniscus progenitors. Here, isolated FN-Prog showed affinity for fibronectin up to at least passage 4. Interestingly, FN-prog from both inner and outer zone meniscus digest formed colonies. The inner zone of the meniscus is regarded as unable to regenerate, but the presence of progenitor-like cells in inner zone meniscus has previously been suggested to contain CD146+ cells [27], clonogenic cells [21], and migrating cells [24,28] with progenitor-like properties. The reason for aberrant regeneration in the inner zone in presence of progenitor cells is unclear, but the lack of contact with blood-derived stimulating factors might prevent the progenitor cells to respond to injury. In agreement with our findings, the number of progenitor-like cells in the inner zone was lower compared to the outer zone in these studies. Therefore, FN-prog from the outer zone are presumably overrepresented in the FN-prog population. Nevertheless, the entire meniscus could be used as a cell source to obtain FN-prog. This facilitates easy isolation and increases the amount of available tissue. Moreover, the presence of FN-prog or progenitor-like cells in the inner meniscus creates potential for repair or regeneration in the inner zone, e.g., by enhancing the activity or density of FN-prog. This could change the current dogma of the inability of inner zone meniscus to regenerate.

The ability to grow clonally is one of the characteristics of progenitor cells. In the current study, FN-prog formed more colonies and proliferated faster than Men. Likewise, a larger colony number and size were previously reported in progenitor-like cells compared to meniscus cells [21,28]. The possibilities for fast and extensive culture expansion creates potential for the use of FN-prog for clinical tissue engineering purposes. The prolonged and fast proliferation might indicate a more progenitor-like state/stemness of this population.

Another indicator of the progenitor-like phenotype of FN-prog is the multilineage potential. The multilineage potential of meniscus progenitor cells was previously reported in progenitor populations selected based on colony formation [21,29,30] or migration from the meniscus [24,28,31]. Here, FN-prog differentiated towards the adipogenic, osteogenic, and chondrogenic lineage, contrary to meniscus cells that did not display chondrogenic differentiation. Here, in contrast to bone-marrow derived MSCs, and similar to results shown for cartilage progenitor cells [20], a lack of hypertrophic differentiation was demonstrated in FN-prog. Again, these characteristics support the use of FN-prog over MSCs for meniscus tissue engineering [11,32].

*COL1A1* and *ACAN* expression of FN-prog were low during expansion and normalized upon redifferentiation, a phenomenon also seen in culture expanded chondrocytes [18,33]. Notably, upon addition of TGF-β to the chondropermissive medium, the *COL2A1* expression was higher in FN-prog than in Men upon culture, although the expression was too low to translate into an abundant deposition of type II collagen on histology. Together with the GAG deposition, this indicates a progenitor-like state with chondrogenic tendency and makes FN-prog a feasible cell type for cartilage tissue engineering. However, only a limited amount of GAGs is found in the healthy native meniscus [34]. Therefore, GAG deposition might be a suboptimal outcome to assess meniscus extracellular matrix formation, and type I collagen deposition could be used instead. Both FN-prog and Men showed type I collagen deposition.

The surface marker profile of FN-prog corresponded largely to the profile for MSC marker expression as defined by the ISCT. High expression of CD90 and CD105 are also found in populations of both progenitor cells and fibrochondrocytes as shown by single cell RNA sequencing at passage 0 [22]. However, apart from the differences in CD73 expression, the markers do not discriminate between Men and FN-prog. Additionally, MSC marker expression increases after culture expansion [23]. The inability to discriminate between cell populations based on immunophenotype is a known drawback in MSC research [35]. To verify the existence of a true and pure meniscus progenitor population, specific markers are currently lacking. More specific markers might increase the purity of this population or demonstrate the physiological or pathological role in the meniscus.

Furthermore, HLA-DR expression was positive in four out of five FN-prog donors. Although MSCs are explicitly defined by negativity for HLA-DR [25], HLA-DR expression has even been found in clinical batches of bone marrow MSCs from two different good manufacturing practice facilities and should not be used as a strict criterion for the release of MSC [36,37]. HLA-DR expression of bone marrow MSCs is upregulated in an inflammatory environment, e.g., by contact with interferon γ [36,38]. Similarly, the HLA-DR expression in meniscus progenitors might be upregulated due to the osteoarthritic inflammatory environment. However, HLA-DR positive and negative MSCs do not differ in morphological, differentiation, and immunomodulatory characteristics. Thus, HLA-DR expression might not be relevant for the MSC function or even improve the anti-inflammatory properties of MSCs [36,38]. Furthermore, expression might be decreased by expansion beyond confluence [39], which might be applied to FN-prog for allogeneic use to decrease the risk of immune reactions. The effect of HLA-DR expression on FN-prog behaviour and the effect of increasing culture time on HLA-DR expression of FN-prog remain to be investigated.

Finally, the progenitor cells were selected by differential adhesion to fibronectin, which has been extensively used for the selection of progenitor cells from articular cartilage [16,17,40,41]. At passage 4, the FN-prog still had more affinity for fibronectin compared to Men. This does imply that the expansion of the total meniscus population does not selectively increase the population of FN-adherent cells and that the FN-prog is a distinctive population. For articular cartilage progenitors, the fibronectin receptor CD49e is responsible for the increased fibronectin adhesion capacity [16]. For these cells, it was shown that the expression of CD49e did not change between passages 0 and 10 [42], but the expression of the fibronectin receptor is dynamic as it increased in culture [43]. In the meniscus, fibronectin is located in the cell membrane of fibrochondrocytes and in the territorial matrix throughout the meniscus [44]. To our knowledge, it is unknown how osteoarthritis influences this distribution. In our current study, fibronectin is only used for the initial selection directly after isolation of the cells from the meniscus. Subsequently, the cells are transferred and passaged on tissue culture plastic without fibronectin coating. Therefore, it is unlikely that the fibronectin adhesion has an influence on the differentiation of the selected cells at passage 4.

### 3.1. Limitations

The cell populations compared in this study are both isolated from OA meniscus, which draws into question the applicability of these cell types for tissue engineering of healthy meniscus. The inflammatory environment of an OA joint might activate degenerative pathways, like the interleukin 1β induced activation of degenerated meniscus progenitor cells [22]. The use of healthy meniscus cells is not a practical alternative, due to the limited availability. Moreover, the degenerative state of meniscus progenitors might be reversible as a shift from degenerative meniscus progenitors to meniscus progenitors was seen upon TGF-β treatment [22]. This identifies TGF-β as a possible treatment target. The use of meniscus fragments obtained during meniscectomy of traumatic meniscus injury might hold potential over the use of osteoarthritic injury. Indeed, in chondrocytes from cartilage, defect rims performed better than chondrocytes from healthy (non-weight baring) regions [45]. However, caution should be exercised until a comparison with populations from healthy meniscus has been made.

### 3.2. Implications

The currently isolated progenitor population is an attractive option for tissue engineering purposes. The availability of FN-prog is almost unlimited as the entire OA meniscus can be used for isolation which is often discarded as redundant material after total knee replacement. Fast expansion and continued in vitro redifferentiation as indicated by type I deposition and proteoglycan production enables large scale (off the shelf) usage. Further research should elucidate the role of FN-prog in healthy and osteoarthritic tissue in order to employ these cells as a therapeutic target or to increase the defective endogenous regeneration and at clinical translation of this cell population.

## 4. Materials and Methods

### 4.1. Cell Isolation and Culture

**Figure 6:**
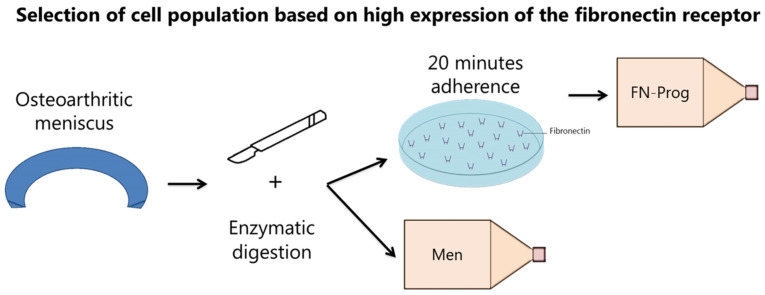
Isolation and selection of cell populations.

Collection of meniscus tissue was performed according to the Medical Ethics regulations of the University Medical Center Utrecht and the guideline “Human Tissue and Medical Research: Code of Conduct for responsible use” of the Dutch Federation of Medical Research Societies [46,47]. Meniscus tissue was obtained from patients with Kellgren and Lawrence grade III and IV osteoarthritis undergoing total knee arthroplasty (*n* = 11, age 52–84). Both female and male donors were used and both donor genders were balanced within experiments. No individual grading was performed on the tissue. Lateral and medial menisci were pooled and cut into cubical pieces of approximately 2 mm^3^ and digested for 2 h in Dulbecco’s modified Eagle medium (DMEM, Gibco, Life Technologies Europe B.V., Bleiswijk, The Netherlands) with 100 U/mL penicillin (Gibco) and 100 µg/mL streptomycin (Gibco) (1% p/s) and 0.2% pronase (Roche Diagnostics GmbH, Mannheim, Germany) under continuous movement (20 rpm) at 37 °C, followed by digestion in DMEM with 1% p/s, 5% heat-inactivated Fetal Bovine Serum (FBS; Biowest, Nuaillé, France) and 0.075% collagenase II (Wortington Biochemical Corporation, Lakewood, NJ, USA). For the total meniscus population (men), the digest was plated on culture flasks and cultured in meniscus expansion medium (DMEM with 10% FBS and 1% p/s) up to passage 2. For the isolation of FN-prog, culture flasks were coated with 10 ng/mL fibronectin (Sigma-Aldrich, Saint Louis, MO, USA) in PBS at 37 °C for 2 h. A total of 500 cells/cm^2^ were plated on the coated flasks and non-adherent cells were removed after 20 min (Figure 6). FN-prog were cultured up to passage 4 in progenitor expansion medium (αMEM (minimal essential medium, Gibco) with 10% FBS, 20 mM l-ascorbic acid-2-phospate (1% ASAP; Sigma-Aldrich) and 5 ng/mL basic fibroblast growth factor (bFGF; Peprotech, London, UK) at 37 °C and 5% CO_2_. For comparison between Men and FN-prog, Men cells were switched to progenitor expansion medium after the first passage and cultured up to passage 4 in progenitor expansion medium. Cells were passaged upon reaching 90% confluency. Population doublings per day were calculated by dividing the number of harvested cells by the number of seeded cells and the number of days. For comparison between inner and outer zones, the outer third and inner third of 5 menisci were digested separately and colony formation was assessed as described below.

### 4.2. Colony Formation

To assess colony formation and affinity for fibronectin, cells were seeded on fibronectin coated wells in a density of 500 cells/cm^2^ (passage 0), 222 cells/cm^2^ (Men passage 2), 111 cells/cm^2^ (Men passage 4), or 22 cells/cm^2^ (FN-prog passage 4). After 20 min, non-adherent cells were removed, and progenitor expansion medium was added. After 3 days, medium was renewed, and after 7 days the cells were fixed and colonies visualized using 0.05% Crystal Violet (Sigma-Aldrich) in Milli-Q water. To assess colony formation in absence of a prior fibronectin adhesion step, 11 cells/cm^2^ (Men passage 2, FN-prog passage 4) and 6 cells/cm^2^ (FN-prog passage 4).

### 4.3. Multilineage Differentiation

For osteogenic differentiation, cells were cultured in monolayer until 50–70% confluent and differentiated for 3 weeks in osteogenic medium (αMEM with 10% FBS, 1% ASAP, 1% p/s, 10 mM β-glycerolphosphate, and 10 nM dexamethasone). For adipogenic differentiation, cells were cultured until confluent and differentiated for 3 weeks in adipogenic medium (αMEM with 10% FBS, 1% p/s, 1µM dexamethasone (Sigma-Aldrich), 0.5 mM isobutylmethylxanthine (Sigma-Aldrich), 0.2 mM indomethacin (Sigma-Aldrich), and 1.72 µM insulin (Sigma-Aldrich)). For chondrogenic differentiation, 250,000 cells were pelleted and cultured for 3 weeks in chondrogenic medium (DMEM, 1% ASAP, 1% p/s, 1% Insulin-Transferrin-Selenium+ Premix (Corning, Corning, NY, USA), 0.1 µM dexamethasone, and 10 ng/mL TGF-β1 (Peprotech)). For hypertrophic differentiation, pellets were cultured for chondrogenic differentiation followed by a 1-week culture in hypertrophic medium (DMEM, 1% ASAP, 1% p/s, 0.2 mM dexamethasone, 10 mM β-glycerolphosphate, and 1 nM triiodothyronine (Sigma-Aldrich)). Following osteogenic differentiation, cells were fixed in 70% ethanol and stained with 40 mM Alizarin Red S (pH 4.1; Sigma-Aldrich) for 5 min. Following adipogenic differentiation, cells were fixed in 4% buffered formaldehyde solution and stained with 0.3% Oil Red O (Sigma-Aldrich) in isopropanol for 30 min. Following chondrogenic and hypertrophic differentiation, pellets were fixated in a 4% buffered formaldehyde solution and further processed as described in ‘histology and immunohistochemistry’.

### 4.4. Expression of MSC Markers

Cells were labeled with antibodies against CD105, CD73 (R&D Systems, Minneapolis, MN, USA), CD90, CD34, CD79A, HLA-DR (Miltenyi Biotec Bergisch, Gladbach, Germany), CD11b, and CD45 (Biolegend, San Diego, CA, USA) according to the manufacturer’s instructions. Cells were mixed with the antibodies in FACS buffer (0.5% bovine serum albumin, 2 mM EDTA in PBS) and incubated in the dark at room temperature for 30 min. Samples were analyzed on a FACSCantoII and LSR Fortessa X20 (BD Biosciences, Allschwil, Switzerland). Stainings with single antibodies and fluorescence minus one were used as controls.

### 4.5. Chondropermissive Cultures/ Redifferentiation

For the analysis of redifferentiation after expansion, 250,000 cells were pelleted and cultured at 37 °C and 5% CO_2_ for 28 days in chondropermissive medium (DMEM, 1% ASAP, 1% p/s, 2% Albuman (Human Serum Albumin, 200 g/L; Sanquin Blood Supply Foundation, Amsterdam, the Netherlands), 2% insulin-transferrin-selenium-ethanolamine (ITS-X; Gibco)) in the absence and presence of 10 ng/mL TGF-β1. Per group, 5 donors were used. Medium was changed twice per week and collected for analysis.

### 4.6. Gene Expression

Gene expression was assessed at the end of the expansion phase and after 28 days of redifferentiation culture. RNA was isolated using TRIzol reagent (Invitrogen, Carlsbad, CA, USA) according to the manufacturer’s recommendations. Then, 200–500 ng RNA was reverse-transcribed using the High-Capacity Reverse Transcription Kit (Applied Biosystems, Foster City, CA, USA). Real-time polymerase chain reactions (RT-PCR) were performed using an iTaq Universal SYBR Green Supermix (Bio-Rad, Hercules, CA, USA) on a LightCycler 96 (Roche Diagnostics) according to the manufacturer’s recommendations. RNA levels were quantified relative to levels of housekeeping gene 18S. Primer sequences can be found in Table 1.

### 4.7. Release and Deposition of Glycosaminoglycans and Collagen

Pellets were harvested after 28 days and digested using a papain digestion buffer (250 µg/mL papain; Sigma-Aldrich, 0.2 M NaH_2_PO_4_, 0.1 M ethylenediaminetetraacetic acid [EDTA], 0.01 M cysteine, pH 6) at 60 °C overnight. GAG content in the digests (deposition) and medium (release) was assessed using a dimethylmethylene blue (DMMB; pH 3) assay to quantify sulphated GAGs. The absorbance was measured at 525 and 595 nm using a spectrophotometer and the ratio at 525/595 nm calculated. Chondroitin-6-sulfate (Sigma-Aldrich) was used as a standard.

For the analysis of collagen deposition, digests were lyophilized followed by a hydrolyzation in 4 M NaOH overnight at 108 °C. Samples were neutralized using 1.4 M citric acid. Then, 50 mM chloramine-T (Merck, Darmstadt, Germany) in oxidation buffer was added. After 20 min incubation, dimethylaminobenzoaldehyde (Merck) in 25% (*w*/*v*) perchloric acid in 2-propanol was added. Absorbance was measured at 570 nm after 20 min incubation at 60 °C. Hydroxyproline (Merck) was used a standard since 13.5% of collagen is composed of hydroxyproline [48]. DNA content of digests was measured using a Quant-iT PicoGreen dsDNA assay (Invitrogen) and was used to normalize collagen and GAG content.

### 4.8. Histology and Immunohistochemistry

Pellets were harvested after 28 days and fixated in a 4% buffered formaldehyde solution. After embedding in paraffin, 5 µm sections were cut. After deparaffinization, sections were stained with 0.4% Fast Green (Merck) followed by 0.125% Safranin-O (Merck) and Weigerts hematoxylin (Clin-Tech, Surrey, UK). Immunohistochemistry for type I and II was performed as following. Antigen were retrieved with 1 mg/mL pronase (Sigma-Aldrich) for 20 min at 37 °C, followed by 10 mg/mL hyaluronidase (Sigma-Aldrich) 20 min at 37 °C. Sections were blocked with a 5% bovine serum albumin (BSA) in PBS solution for 30 min. Samples were incubated with the primary antibody (type I collagen, rabbit monoclonal 1/400 or type II collagen, mouse monoclonal 1/100 in PBS/BSA 5%) overnight at 4 °C. Sections were washed and incubated with horseradish peroxidase-conjugated anti-rabbit or mouse secondary antibody (Dako, Glostrup, Denmark) for 30 min at room temperature. For type X collagen, antigen were retrieved using 1 mg/mL pepsin (Sigma-Aldrich) in 0.5M acetic acid for 2 h at 37 °C, followed by 10 mg/mL hyaluronidase for 30 min at 37 °C. Sections were blocked with 5% BSA in PBS for 30 min. Samples were incubated with the primary antibody (type X collagen, mouse monoclonal 1/20 in PBS/BSA 5%) overnight at 4 °C. Sections were washed and incubated with biotin-conjugated anti-mouse secondary antibody (GE healthcare, Little Chalfont, UK) for 60 min at room temperature, followed by an enhancement step with streptavidin—peroxidase (Beckman Coulter, Woerden, The Netherlands) for 60 min at room temperature. Immunoreactivity was visualized using diaminobenzidine peroxidase substrate solution (DAB, Sigma-Aldrich). Mayer’s hematoxylin was used as counterstaining.

### 4.9. Statistical Analyses

Statistical analyses were performed using GraphPad Prism 8.3 (GraphPad Software, Inc., La Jolla, CA, USA). Data are presented as mean ± standard deviation (SD). Colony formation was compared between inner and outer zone of the same donor using a two-tailed paired t-test (Figure 1a). To test for differences in colony formation and population doubling between FN-prog and Men, a one-way ANOVA with Dunnett’s multiple comparisons correction was performed (Figure 1b–d). Relative gene expression of FN-prog at passage 4 was compared to the expression Men at passages 2 and 4 using a one-way ANOVA with Dunnett’s multiple comparisons correction (Figure 3a,b). To assess differences in CD318 and MCAM surface marker expression between Men and FN-prog of the same donors, a two-tailed paired t-test was used (Figure 3C). Relative gene expression and matrix formation of Men and FN-prog were compared in the presence and in absence of TGF-β1 using a one-way ANOVA with Dunnett’s multiple comparisons correction (Figure 4a,b). *p* values below 0.05 were considered significant.

## Figures and Tables

**Table 1:** Primer sequences for quantitative real-time PCR. ACAN, aggrecan; bp, base pair; COL1A1, collagen type I alpha 1 chain; COL2A1, collagen type II alpha 1 chain; CDK1, Cyclin-dependent kinase 1; DNER, Delta and Notch-like epidermal growth factor-related receptor; Fw, forward; GREM1, Gremlin-1; MCAM, Melanoma cell adhesion molecule; Rv, Reverse.

Gene Name	Oligonucleotide Sequence (5′ to 3′)	Product Size (bp)
18S	Fw: GTAACCCGTTGAACCCCATT Rv: CCATCCAATCGGTAGTAGCG	151
ACAN	Fw: CAACTACCCGGCCATCC Rv: GATGGCTCTGTAATGGAACAC	160
COL2A1	Fw: AGGGCCAGGATGTCCGGCA Rv: GGGTCCCAGGTTCTCCATCT	195
COL1A1	Fw: TCCAACGAGATCGAGATCC Rv: AAGCCGAATTCCTGGTCT	191
DNER	Fw: AAGGCTATGAAGGTCCCAACT Rv: CTGAGAGCGAGGCAGGATTT	137
MCAM	Fw: AGCTCCGCGTCTACAAAGC Rv: CTACACAGGTAGCGACCTCC	102
CDK1	Fw: AAACTACAGGTCAAGTGGTAGCC Rv: TCCTGCATAAGCACATCCTGA	148

## Data Availability

The data presented in this study are available on request from the corresponding author.
